# Assessment of HIPIMS-Deposited TiN Nanostructured Thin Films as Hydrogen Permeation Barriers on Carbon Steel

**DOI:** 10.3390/ma19081623

**Published:** 2026-04-17

**Authors:** Raúl González-Durán, Alvaro Rodríguez-Prieto, Ana María Camacho

**Affiliations:** Department of Manufacturing Engineering, National University of Distance Education UNED, 28040 Madrid, Spain; alvaro.rodriguez@ind.uned.es (A.R.-P.); amcamacho@ind.uned.es (A.M.C.)

**Keywords:** hydrogen nitride (TiN), PVD coating, high power impulse magnetron sputtering (HIPIMS), materials performance, hydrogen permeation

## Abstract

Hydrogen embrittlement (HE) represents a critical degradation mechanism in carbon steel components operating in hydrogen-rich environments, such as those encountered in clean energy and petrochemical applications. This study evaluates the hydrogen permeation barrier performance of titanium nitride (TiN) nanostructured thin films deposited by High-Power Impulse Magnetron Sputtering (HiPIMS) on SAE 1020 carbon steel substrates. Electrochemical permeation measurements were performed using the Devanathan–Stachurski dual-cell methodology in accordance with ASTM G148 and ISO 17081 standards. Key hydrogen transport parameters quantified include the effective diffusion coefficient (D_eff_), lag time (t_lag_), and steady-state hydrogen oxidation current density. The TiN/carbon steel composite system exhibited t_lag_ = 570 s, D_eff_ = (2.68 ± 0.09) × 10^−10^ m^2^ s^−1^ and a steady-state hydrogen oxidation current density of 21.5 µA cm^−2^, corresponding to a permeation reduction factor (PRF) of 2.32 and a barrier efficiency of η = 56.9%. The superior barrier performance is attributed to the dense, low-defect microstructure characteristic of HiPIMS deposition. These results validate HiPIMS-deposited TiN as a robust hydrogen diffusion barrier, with the established performance metrics providing quantitative benchmarks for the design of hydrogen-resistant coatings in energy applications.

## 1. Introduction

The global energy transition from fossil fuel to renewables and hydrogen is driven by a synergistic combination of environmental imperatives and economic strategic considerations, which have gained substantial international momentum in recent years [1].

However, materializing a viable hydrogen economy necessitates the development of inherently safe infrastructure and advanced asset management protocols. In this context, hydrogen embrittlement (HE) in metallic structures emerges as a critical degradation mechanism, requiring rigorous material selection, a specialized barrier, and precise design solutions to ensure long-term structural integrity. Given the stochastic and multi-scale complexity of HE mechanisms, a profound understanding of the underlying kinetic and thermodynamic processes is essential to predict and mitigate catastrophic fractures in metallic systems [2]. Moreover, hydrogen systems must withstand high-pressure conditions for extended periods. Under such demanding service environments, hydrogen-related failures particularly material embrittlement compromise the reliability of industrial components and, consequently, hinder the sustainable development of the hydrogen energy sector [3].

Hydrogen-induced degradation in metallic materials typically originates from environmental exposure. Specifically, the process is initiated when H_2_ molecules adsorb onto the metal surface, dissociate into atomic hydrogen, and subsequently diffuse into the bulk material. Consequently, it is of fundamental importance to understand both the role of hydrogen in embrittlement and its complex interactions with microstructural defects. While hydrogen atoms in solid solution—occupying interstitial lattice sites—often represent only a minor fraction of the total hydrogen content under thermal equilibrium, these sites are numerically the most abundant. As a result, they serve as the primary medium for hydrogen transport and effectively govern how hydrogen is partitioned among various trapping sites throughout the microstructure [4].

Several mechanisms have been proposed to explain hydrogen-induced fracture. The Hydrogen-Enhanced Decohesion (HEDE) theory postulates that dissolved hydrogen reduces the cohesive strength of metallic bonds at the atomic level, leading to microvoid formation and brittle fracture, typically intergranular in nature. In contrast, the Hydrogen-Enhanced Localized Plasticity (HELP) theory attributes embrittlement to increased dislocation mobility caused by hydrogen diffusion, which results in localized plastic deformation and early crack initiation. More recently, the Absorption-Induced Decohesion Emission (AIDE) theory has been proposed, suggesting that absorbed hydrogen weakens atomic bonding forces, leading to decohesion and microstructural separation [5].

To mitigate these adverse effects, surface engineering approaches such as titanium nitride (TiN) nanostructured thin films have shown significant promise. TiN coatings, typically deposited by Physical Vapor Deposition (PVD) techniques, particularly High-Power Impulse Magnetron Sputtering (HIPIMS), provide dense, adherent, and diffusion-resistant barriers capable of reducing hydrogen ingress. HIPIMS technology enables high ionization of the target material through high-power pulsed discharges, producing coatings with superior microstructure densification and improved resistance [6].

Beyond monolithic TiN, other Nitride-based ceramics such as Boron Nitride (BN) are critical for mitigating hydrogen permeation in metallic substrates. While TiN-TiC composites offer exceptional adhesion and can reduce hydrogen permeability by up to four orders of magnitude. Advanced TiO_2_-TiN-TiC triple-layer architectures address these gaps by providing higher density and stability. Ultimately, the success of BN coatings depends on phase selection, ranging from the ultra-hard c-BN to the graphene-like h-BN.

Regarding the quantification of hydrogen transport, precise experimental methodologies are required to evaluate these barriers. While gas-phase permeation is a common approach, it often necessitates elevated temperatures (>200 °C for steel membranes), which can alter trap-site equilibrium and limit its applicability for room-temperature studies. In contrast, this study assesses the hydrogen barrier performance of TiN coatings using standardized electrochemical permeation methods in accordance with ASTM G148 and ISO 17081. through a Devanathan–Stachurski cell configuration, This approach allows for the accurate determination of the hydrogen diffusion coefficient and permeation flux, providing a quantitative measure of coating efficiency under ambient conditions [7,8,9].

The primary objective of this research is to evaluate the diffusion behavior of hydrogen through nanostructured TiN thin films deposited by HIPIMS and to identify the limitations and protective efficiency of such coatings in preventing hydrogen ingress.

Consequently, this work addresses critical challenges associated with hydrogen production, transportation, and storage by integrating electrochemical theory, materials science, and the mechanics of hydrogen-induced damage. By contributing to the understanding of hydrogen–coating interactions, this study aims to support the development of reliable mitigation strategies within the framework of Asset Integrity Management (AIM) for critical energy infrastructure. As global energy demand continues to rise, diversifying energy sources becomes essential to ensure energy security and sustainability. Therefore, hydrogen is expected to play a pivotal role in the future energy mix, making the development of durable and hydrogen-resistant materials a key enabler for a sustainable hydrogen economy.

## 2. Materials and Methods

### 2.1. Substrate Material and Specimen Preparation

The substrate material was SAE 1020 hot-rolled carbon steel, with a nominal composition of 0.18–0.23 wt.% C, 0.30–0.60 wt.% Mn, ≤0.030 wt.% P, ≤0.035 wt.% S, and balance Fe. This alloy, in the as-received hot-rolled condition without subsequent heat treatment, is a hypoeutectoid steel whose room-temperature microstructure consists predominantly of ferritic (α-iron) grains with minor pearlite colonies.

Specimens were sectioned into square coupons measuring 10 × 10 × 1 mm, with a final verified thickness of 0.97 ± 0.01 mm as determined by digital micrometry (resolution ±0.01 mm). Prior to coating deposition, specimens underwent systematic surface preparation consisting of mechanical grinding with successive silicon carbide abrasive papers from P120 to P1200 grit, followed by diamond-paste polishing to a mirror finish (Ra < 0.1 μm, confirmed by contact profilometry). Specimens were subsequently ultrasonically cleaned in acetone for 10 min, rinsed in ethanol for 10 min, and dried under a stream of high-purity nitrogen. No palladium coating was applied to the detection (oxidation) side, in accordance with the bare-steel detection surface requirements specified in ASTM G148 [8] for ferritic substrates.

### 2.2. TiN Coating Deposition

Titanium nitride (TiN) thin films were deposited onto the face of prepared carbon steel coupons using a High-Power Impulse Magnetron Sputtering (HIPIMS) system equipped with a high-purity titanium target. To ensure optimal coating properties, the deposition was carried out in a nitrogen/argon (N_2,_ up to 10 sccm/Ar, up to 40 sccm), atmosphere and Pulse duration (0–200 μs). Regarding parameters, the HIPIMS power supply operated at a pulse frequency of up to 1000 Hz and reached a peak power density of up to 10 kW/cm^2^. During this process, the plasma consisted of both gas ions (argon and nitrogen) and titanium metal ions. Consequently, the high ionization of the film-forming species promoted the growth of a dense microstructure, which is characteristic of high-quality HIPIMS coatings. Finally, the resulting films exhibited a distinctive golden hue, providing a visual indication of proper stoichiometric TiN formation.

### 2.3. Electrochemical Hydrogen Permeation Measurements

Hydrogen permeation measurements were conducted using a Devanathan–Stachurski (D–S) dual-cell electrochemical apparatus integrated with a bipotentiostat system, with the entire experimental methodology designed in strict accordance with international standards ASTM G148 [8] and ISO 17081 [9].

Detection Side Stabilization: The detection compartment is filled with 0.1 M NaOH and a Potentiostatic potential is applied until the background current indicates a passivated surface ready for hydrogen detection.

The electrochemical cell comprises two distinct compartments separated by TiN-coated specimen, which functions simultaneously as the working electrode and the permeation membrane. Specifically, the charging compartment (cathodic side) exposes the TiN-coated surface to the electrolyte, whereas the detection compartment (anodic side) exposes the uncoated steel surface. Each compartment is equipped with a platinum mesh counter electrode and an Ag/AgCl reference electrode. Furthermore, the bipotentiostat enables independent yet simultaneous control of the electrochemical conditions on both sides of the membrane, thereby ensuring precise decoupling of hydrogen entry and exit kinetics.

The hydrogen permeation test procedure was executed through three sequential stages. In the first stage, hydrogen charging was performed on the TiN-coated surface. The charging compartment was filled with a deaerated aqueous solution of 0.1 M H_2_SO_4_ containing thiourea (CH_4_N_2_S) as a hydrogen recombination poison.

In the second stage, the detection side was stabilized. The detection compartment was filled with deaerated 0.1 M NaOH, and a potentiostatic potential of +300 ± 5 mV versus open-circuit potential was applied to the uncoated steel surface until the background current stabilized below 0.1 μA/cm^2^, indicating the establishment of a stable passive film and surface readiness for hydrogen detection.

In the final stage, permeation recording was initiated. The hydrogen permeation flux was continuously recorded on the detection side as a function of time at a sampling frequency of 1 Hz. Given that the anodic current is directly proportional to the hydrogen flux through the membrane under the applied potentiostatic conditions, this methodology enables quantitative determination of hydrogen transport parameters, including the diffusion coefficient and steady-state permeation flux, at room temperature (20.0 ± 0.5 °C).

**Figure 1:**
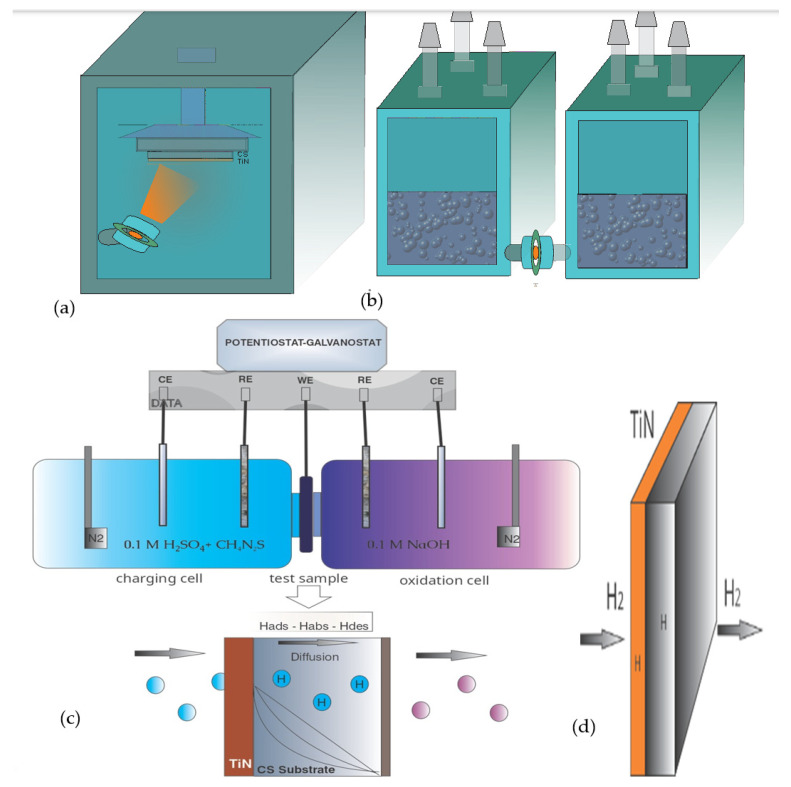
(**a**) Schematic diagram of the HIPIMS deposition system, (**b**) Devanathan–Stachurski (D–S) dual-cell apparatus for hydrogen permeation evaluation. (**c**) Schematic of the D–S electrochemical cell assembly, with bipotentiostat connections. (**d**) Cross-sectional schematic of the TiN/CS bilayer membrane illustrating the hydrogen permeation.

Figure 1 presents a comprehensive schematic illustration of the experimental apparatus employed in this study. Specifically, Figure 1a depicts the High-Power Impulse Magnetron Sputtering (HIPIMS) deposition chamber, which enables the fabrication of dense, uniform titanium nitride (TiN) thin-film coatings on carbon steel (CS) substrates through high-ionization plasma processing. Furthermore, Figure 1b illustrates the Devanathan–Stachurski (D–S) dual-cell apparatus utilized for hydrogen permeation evaluation, whereas Figure 1c provides a detailed schematic of the electrochemical cell assembly. This assembly comprises Ag/AgCl reference electrodes (RE), platinum mesh counter electrodes (CE), and the thin metallic specimen positioned between the two cells, which function simultaneously as the working electrode (WE) and the permeation membrane. Additionally, Figure 1c includes the measuring apparatus configuration and a schematic representation of the sequential processes of hydrogen adsorption, diffusion, and desorption occurring across the membrane [10]. Finally, Figure 1d, shows a Cross-sectional schematic of the TiN/CS bilayer membrane illustrating the hydrogen permeation mechanism and subsurface accumulation within the TiN/steel bilayer system.

### 2.4. Experimental Setup

From the hydrogen permeation, critical parameters were determined:

Hydrogen permeation flux (J) (mol m^−2^ s^−1^), derived from Fick’s first (Equation (1)) and second (Equation (2)) laws [11].

(1) $$J=-D_{eff}\frac{\partial c}{\partial x}$$

(2) $$\frac{\partial C}{\partial t}=D_{eff}\frac{\partial^{2}c}{\partial x^{2}}$$

Effective diffusion coefficient, D_eff_, C the concentration of diffusible hydrogen and x the distance vertical to the surface; thus, dc/dx is the gradient of the hydrogen concentration in the permeation direction in the metal membrane, and t is the time [10,11].

Time-dependent atomic hydrogen permeation flux as measured on the oxidation side of the sample (J(t)) and Atomic hydrogen permeation flux at steady-state as measured on the oxidation side of the sample (Jss), standards ASTM G148 [8] and ISO 17081 [9].

○ Time Lag (*t_lag_*): Defined as the time to achieve a value of Normalized flux of atomic hydrogen J(t)/Jss = 0.63 (s) according standards ASTM G148 [8] and ISO 17081 [9] Equation (3) [10,11].

(3) $$t_{lag}=\frac{L^{2}}{6D}$$

○ Effective Diffusion Coefficient (*D_eff_*): Effective diffusion coefficient of atomic hydrogen based on elapsed time corresponding to *J*(*t*)/*J*ss = 0.63 Equation (4) [11].

(4) $$D_{eff}=\frac{L^{2}}{6t_{lag}}$$

○ Permeation Reduction Factor (*PRF*): A measure of the coating’s effectiveness, calculated as the ratio of hydrogen flux through the bare substrate to that through the coated substrate Equation (5) [11,12].

(5) $$PRF=\frac{J_{uncoated}}{J_{coated}}$$

○ Barrier Efficiency (η) Equation (6):

(6) $$\eta (\%)=1-\frac{1}{PRF}\times 100$$

Table 1 describes the experimental matrix conditions for TiN on CS materials and defines the electrolyte composition.

## 3. Results

**Figure 2:**
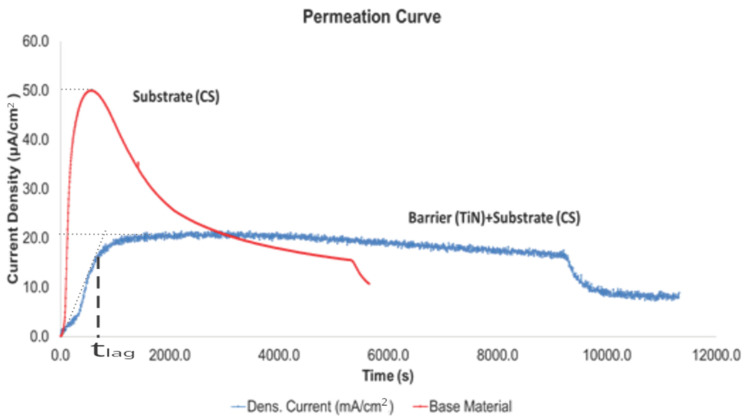
Hydrogen permeation results in curves of coating + substrate vs. substrate; determination of t_lag_.

The results of the hydrogen permeation assessment are displayed in Figure 2, which includes the coating + substrate vs. substrate slope curves, and identifies the (t_lag_), steady-state current density (Jss), and the peak current density.

### 3.1. Hydrogen Permeation Evaluation

#### 3.1.1. Hydrogen Permeation Transients

Figure 2 presents the hydrogen oxidation current density (µA cm^−2^) as a function of time for the uncoated SAE 1020 reference (CS, red) and HIPIMS-TiN/CS composite (blue), under identical charging conditions (Jc = 7.291 mA cm^−2^, T = 20.0 ± 0.5 °C; N = 1 per condition).

#### 3.1.2. Permeation Behavior of Uncoated Carbon Steel (Reference Specimen)

The permeation transient for the uncoated steel (Figure 2, red curve) demonstrates high hydrogen permeability, marked by an almost instantaneous current increase upon cathodic charging. Reaching a peak of 50 μA/cm^2^, this rapid response confirms efficient transport through the 0.97mm membrane, yielding a diffusion coefficient of 1.03 × 10^−9^ m^2^/s, For the uncoated CS specimen, true steady state was not attained within the 12,000 s experiment; The permeation current reached a peak at approximately 500 s then declined. D_eff,CS_ was estimated from the pre-peak rising segment using the time-lag method: t_lag,CS_ ≈ 153 s (identified at Jox(t)/Jox, peak = 0.63 on the rising portion). D_eff,CS_ = L^2^/(6 × t_lag,CS_) = (0.97 × 10^−3^)^2^/(6 × 153) = 9.409 × 10^−7^/918 = 1.03 × 10^−9^ m^2^s^−1^) [11].

#### 3.1.3. Permeation Evaluation Curves Behavior of TiN-Coated Carbon Steel (Barrier Specimen)

In contrast, the TiN-coated specimen (blue curve, Figure 2) shows markedly better barrier performance than the uncoated steel, with three distinguishing features. First, it exhibits a pronounced lag time (t_lag_) of 570 s, during which the permeation current stays near background level, indicating that the coating delays hydrogen entry into the steel. Second, after breakthrough the current rises more slowly and reaches a quasi-steady-state plateau at a limiting current density (Isat) of 21.5 μA/cm^2^, well below the uncoated sample’s peak. Third, the steady-state flux is about 56% lower than the uncoated steel’s 50 μA/cm^2^ peak. This reduction means considerably less hydrogen permeates per unit time, thereby lowering the risk of hydrogen embrittlement under the applied charging conditions.

### 3.2. Quantification of Hydrogen Diffusion Parameters

From the experimental permeation transients obtained for both specimens, a set of characteristic diffusion parameters was derived, as detailed in Table 2 and Table 3. For the TiN-coated sample, the lag time (t_lag_) of 570 s, in conjunction with the membrane thickness (L = 0.97 mm), generated an apparent effective diffusion coefficient of D_eff_ = 2.68 × 10^−10^ m^2^ s^−1^, calculated according to the standard lag-time method. It is important to emphasize that this value does not represent the intrinsic diffusion coefficient of TiN itself; rather, it reflects the effective transport parameter of the composite TiN/steel system, which incorporates the strong retarding influence of the thin coating layer on the overall hydrogen diffusion kinetics.

Additionally, the limiting current density (Isat) of 21.5 μA cm^−2^ was used to estimate the hydrogen saturation concentration at the entry surface (Csat) under the applied charging conditions, yielding a value of 8.063 mol H m^−3^. Further time-domain parameters extracted from the rising anodic transient include the inflection point time (ti), the half-rise time (t_1/2_), defined as the time required for the permeation current to reach half of its steady-state value, and the breakthrough time (tb), obtained by extrapolating the tangent at the inflection point to the baseline current level [9]. These parameters collectively provide a comprehensive characterization of the hydrogen transport kinetics within the coated system.

**Figure 3:**
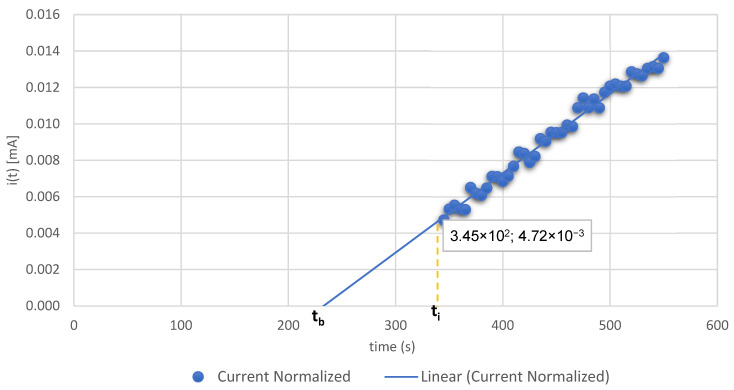
Hydrogen permeation results in curves of coating + substrate slope; determination of t_b_ and t_i_.

In summary, these results clearly demonstrate that a thin TiN layer deposited by High-Power Impulse Magnetron Sputtering (HiPIMS) profoundly modifies the hydrogen permeation response of carbon steel. The coating acts as a highly effective diffusion barrier, simultaneously delaying the onset of hydrogen ingress and reducing the steady-state permeation flux by more than 50%, thus validating its application as a robust and practical strategy for mitigating hydrogen embrittlement in structural steel components. The corresponding permeation curves and derived parameters are presented in Figure 2 and Figure 3 and Table 2 and Table 3, respectively.

Figure 3 shows the results of the hydrogen permeation evaluation results in curves of coating + substrate slope; determination of Elapsed time measured by extrapolating the linear portion of the rising permeation current transient (t_b_) and inflection time (t_i_).

To provide a structured and quantitative assessment of the protective performance of the TiN coating, the principal hydrogen permeation parameters derived from the experimental transients are compiled in Table 2. The comparative evaluation encompasses six analytical descriptors, each of which captures a distinct and complementary aspect of hydrogen transport behavior across the uncoated SAE 1020 carbon steel substrate (CS) and the TiN-coated specimen (TiN/CS).

The calculated Permeation Reduction Factor (PRF) peak of approximately 2.32 (50 µA/cm^2^ and 21.5 µA/cm^2^), (PRF)ss of approximately 1.09 (23.5 µA/cm^2^ and 21.5 µA/cm^2^), quantitatively demonstrate the significant reduction in hydrogen permeation achieved by the TiN coating.

Finally, the barrier efficiency (η), defined as the percentage reduction in steady-state permeation flux relative to the uncoated reference, was determined to be approximately 56% for the TiN-coated specimen. This parameter provides a single, unambiguous metric that integrates all the above transport descriptors into a concise measure of the coating’s overall effectiveness. The results are presented in Table 2.

In Table 2 an explanation of results of curves of Figure 2 and Figure 3 is presented. Comparative hydrogen permeation parameters for the uncoated SAE 1020 carbon steel (CS) and the TiN-coated specimen (TiN/CS).

Table 3 presents the comprehensive results obtained from the electrochemical permeation experiments conducted on the TiN-coated test sample under a constant charging current density of 7.291 mA cm^−2^, which drove hydrogen entry into the material. The hydrogen permeation was monitored continuously by measuring the anodic current on the detection side, and a stable background current was systematically recorded prior to initiating cathodic charging.

The calculated values for the diffusion coefficient demonstrate remarkable consistency across the four independent calculation methods employed. Consequently, the average apparent diffusion coefficient was determined to be D_eff_ = (2.68 ± 0.09) × 10^−10^ m^2^ s^−1^, where the narrow range of individual values (coefficient of variation < 4%) indicates high experimental reliability and methodological robustness. Furthermore, the steady-state permeation flux was characterized by a limiting current density of Isat = 0.0215 ± 0.0012 mA cm^−2^, which corresponds to a subsurface hydrogen concentration of Csat = 8.063 ± 0.45 mol H m^−3^ (equivalent to 1.02 ± 0.06 ppm by mass) at the entry side of the material under the specified charging conditions.

The experiment successfully quantified the key hydrogen transport properties, thereby providing crucial data regarding diffusion kinetics and hydrogen solubility in the TiN/steel composite system.

The results of the diffusion parameter calculations and the analyses of Figure 2 and Figure 3 are summarized in Table 3.

### 3.3. Surface Morphology Composition

**Figure 4:**
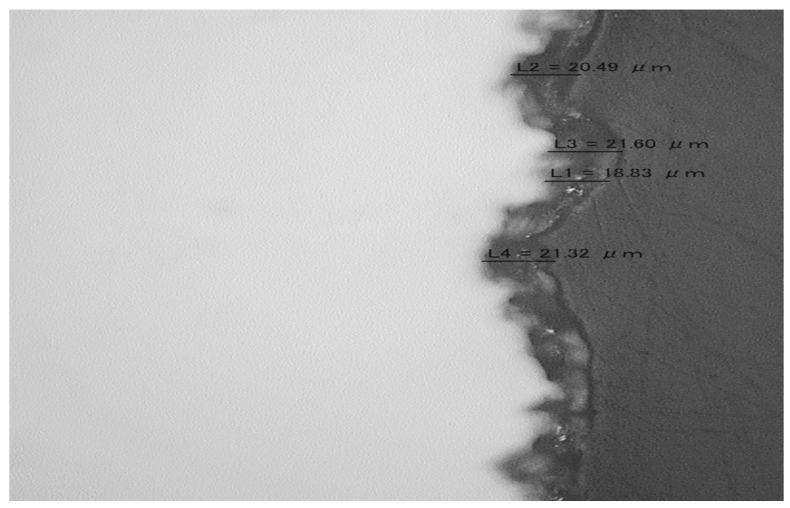
Characterization test sample: Comparison of TiN on CS thickness. High-resolution cross-sectional TEM recommended as future work.

Morphology evaluations of transversal cut and the external surface of test sample by Hirox microscope at 700× and visual inspection were obtained as follows in Figure 4.

Investigations have established that hydrogen permeation is substantially governed by the solid-state diffusion of hydrogen through protective surface films. Consequently, elucidating the complex relationship between diffusion kinetics and surface reactions is imperative for the accurate prediction of diffusion behavior in hydrogen-rich environments [13].

**Figure 5:**
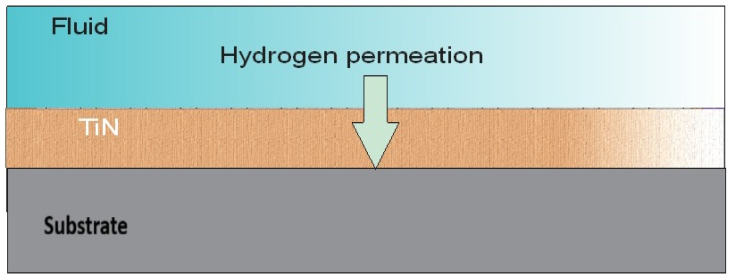
Hydrogen permeation in TiN HIPIMS thin film; TiN/CS.

In the context of the present study, the operative permeation mechanisms through the TiN/carbon steel composite system are schematically illustrated in Figure 5. The Figure describes the hydrogen permeation pathway through an intact TiN thin film deposited on carbon steel, wherein atomic hydrogen must traverse the crystalline TiN layer which presents a significantly lower diffusivity compared to the steel substrate before reaching the detection side of the membrane.

**Figure 6:**
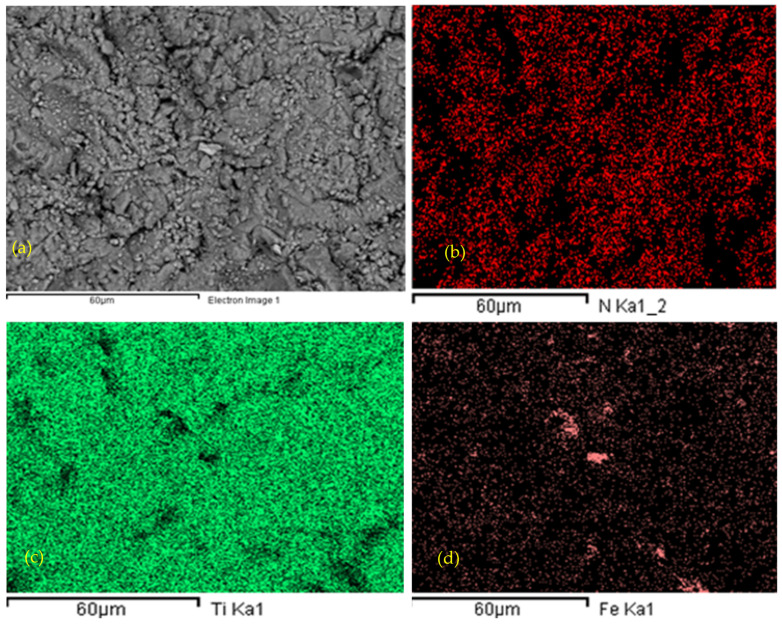

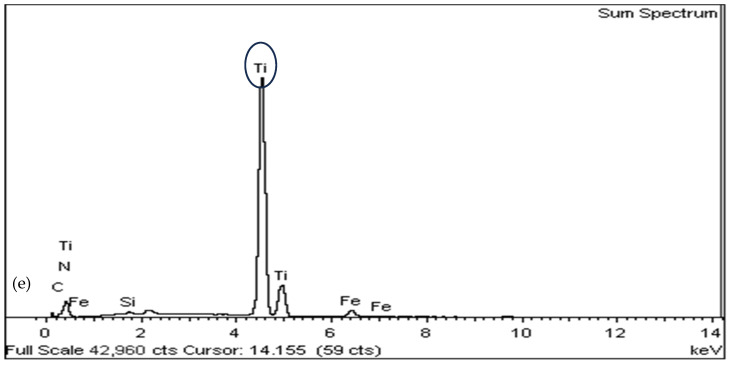
SEM–EDS, characterization of the thin TiN coating on carbon steel plane section, top view (**a**) 1000 × TiN, thin film (**b**) Morphological analysis by SEM–EDS Nitrogen Mapping; (**c**) Morphological analysis by SEM–EDS, top view Titanium Mapping, (**d**) Morphological analysis by SEM–EDS top view Iron Mapping. (**e**) EDS microscopy of a cross view of the thin TiN layer on CS. The NKα mapping is presented as indicative only.

To corroborate the morphological findings and assess the chemical integrity of the deposited layer, elemental mapping via Energy Dispersive X-ray Spectroscopy (EDS) was conducted. Figure 6 presents the EDS mapping analysis of the TiN thin film deposited on the carbon steel substrate. The elemental distribution maps (Figure 6b–d) reveal a homogeneous and uniform distribution of titanium (Ti) and nitrogen (N) across the entire analyzed surface, confirming the successful deposition of a stoichiometrically consistent TiN layer (EDS contribution as qualitative spatial mapping only).

Furthermore, the mapping highlights a distinct and well-defined interface between the coating and the substrate. The absence of significant iron (Fe) signals within the coating matrix indicates a dense, defect-free structure with negligible interdiffusion of substrate elements into the film. This compositional uniformity is critical, as any chemical discontinuity or oxidation could act as a preferential pathway for hydrogen diffusion, thereby compromising the barrier performance. The EDS spectrum (Figure 6e) further validates these observations qualitative, displaying dominant peaks for Ti and N, with trace amounts of other elements attributable to the substrate or surface contamination.

Complementary microstructural and compositional characterization of the TiN thin film was performed by scanning electron microscopy (SEM) coupled with energy-dispersive X-ray spectroscopy (EDS), and the results are presented in Figure 6. Figure 6a–d show plan-view SEM micrographs. Figure 6e presents the qualitative EDS point spectrum acquired from the coating surface of the TiN phase under the employed HiPIMS deposition conditions contribution as qualitative spatial mapping only.

## 4. Discussion

The comparative analysis of the hydrogen permeation transients recorded for the uncoated carbon steel substrate (CS) and the TiN-coated composite system (TiN/CS) reveals both the multifunctional character and the high effectiveness of the deposited coating as a hydrogen diffusion barrier. A detailed interpretation of the curves presented in Figure 2 and Figure 3, in conjunction with the experimentally derived parameters summarized in Table 2 and Table 3, allows for the identification and mechanistic clarification of the operative barrier processes governing hydrogen transport through each system.

### 4.1. Interpretation of Permeation Curve Dynamics

Each morphological feature of the permeation transients carries a well-defined physical significance that is directly related to the underlying hydrogen transport mechanisms. Accordingly, a systematic feature-by-feature analysis is presented below.

#### 4.1.1. Rise Rate and Permeation Kinetics

For the uncoated carbon steel (CS), the near-vertical slope of the rising portion of the permeation transient (Figure 2, red curve) is indicative of extremely rapid permeation kinetics. This behavior aligns with the high intrinsic diffusivity of hydrogen within the ferrite (α-Fe) microstructure of carbon steel. At room temperature, this stable phase has a body-centered cubic (BCC) crystal structure, which facilitates rapid atomic transport. Consequently, in the absence of a surface barrier, the rate-limiting step is governed solely by diffusion through the steel bulk—a process that is inherently efficient in the α-iron lattice [14].

In contrast, The TiN-coated specimen exhibits permeation kinetics dominated by the resistance of the outer layer, whose diffusion coefficient is several orders of magnitude lower than that of the steel. TiN coatings deposited on carbon steel fundamentally alter hydrogen permeation kinetics, shifting the rate-limiting step from diffusion within the metallic substrate to transport through the ceramic overlayer. The TiN layer reduces the effective diffusion coefficient by several orders of magnitude, producing a characteristic lag time of 570 s and lowering the steady-state permeation current to less than half that of bare steel. This barrier efficiency is further enhanced by nanocrystalline microstructures, where grain boundaries act as reversible hydrogen traps, yielding permeation reduction factors (PRF) of 100–5000. Additionally, the Fe/TiN interface—characterized by generates a dense network of misfit dislocations that serve as trapping sites with binding energies of 15–30 kJ/mol. These synergistic mechanisms, including TiN’s inherently slow diffusion, grain-boundary trapping, and interfacial segregation, give the TiN/steel system its superior ability to inhibit hydrogen embrittlement in critical energy infrastructure [15,16,17].

#### 4.1.2. Peak Current Density and Hydrogen Trapping

The permeation transient of the uncoated carbon steel displays a pronounced current density peak of approximately 50 μA cm^−2^, followed by a gradual decay toward a lower steady-state value. This peak can be attributed to the progressive saturation of reversible microstructural trapping sites within the steel including grain boundaries, dislocations, and non-metallic inclusions that temporarily immobilize diffusing hydrogen atoms. In the initial stage, a fraction of the permeating hydrogen is captured by these traps, so that the flux reaching the detection side is transiently suppressed. As the trapping sites approach saturation, the freely diffusing hydrogen flux increases rapidly, culminating in the observed current peak. Once a dynamic equilibrium between trap occupation and detrapping is established, the permeation current stabilizes at its steady-state value.

For the TiN-coated specimen, the absence of an analogous peak in the permeation transient is highly revealing. The hydrogen flux imposed on the steel substrate by the TiN-filtered entry is sufficiently low and slow that the dynamics of trap saturation within the steel are either entirely suppressed or developed on a time scale far exceeding that of the experiment. Under these conditions, the barrier effect of the TiN coating completely dominates the transient response, preventing the accumulation of hydrogen at trapping sites and masking any associated peak current feature.

#### 4.1.3. Steady-State Permeation Current Density

The steady-state permeation current density (Iss) represents the constant hydrogen flux established once a linear concentration gradient is fully developed across the membrane thickness, in accordance with Fick’s first law. The fact that the steady-state current for the TiN-coated sample (Isat = 21.5 μA cm^−2^) is less than half of the corresponding peak value for the uncoated steel demonstrates that the TiN coating not only delays the onset of hydrogen permeation but also permanently reduces the total hydrogen flux that can traverse the membrane under the imposed experimental conditions. This persistent flux reduction constitutes direct evidence of the coating’s sustained barrier action throughout the entire duration of the electrochemical charging experiment.

### 4.2. Correlation with TiN Barrier Mechanisms

The barrier functionality of the HiPIMS-derived TiN coating is defined by its ability to retard hydrogen ingress and attenuate the steady-state permeation flux. These effects are underpinned by distinct but synergistic physicochemical mechanisms.

#### 4.2.1. Lag Time Enhancement and Intrinsic Low Permeability

The extended lag time of 570 s observed for the TiN/CS system originates from two fundamental contributions. First, TiN is a transition metal nitride with a dense, face-centered cubic crystal structure that provides inherently low solubility and diffusivity for hydrogen, both of which are orders of magnitude below the corresponding values for α-iron. This intrinsic low permeability renders the TiN layer a highly effective physical tortuous barrier that drastically slows the diffusion of atomic hydrogen across its thickness. Second, the TiN/steel interface constitutes a region of elevated defect density, arising from lattice parameter mismatch and the presence of misfit dislocations.

#### 4.2.2. Steady-State Flux Reduction

By restricting the rate at which hydrogen atoms traverse the TiN layer, the coating reduces the effective hydrogen concentration at the TiN/steel interface relative to the charging-side concentration. The choice of HiPIMS as the deposition technique is of critical importance in this context since this technology produces coatings with high packing density. These microstructural attributes are essential for ensuring that the permeation resistance of the coating is uniformly distributed and not undermined by localized short-circuit diffusion pathways.

### 4.3. Impact of Experimental Variables and System Stability

The reliability and reproducibility of the permeation measurements depend critically on the precise control of the experimental variables whose values are reported in Table 3. Each parameter applies a distinct influence on the transport properties measured and must therefore be considered when interpreting the results.

The membrane thickness (L) enters the calculation of the effective diffusion coefficient (D_eff_) as a squared term (L^2^), so that even small errors in its measurement propagate significantly into the derived diffusivity value. The 0.97 mm substrate thickness was therefore determined with high precision. Although the TiN coating thickness is substantially smaller, it is primarily responsible for the observed barrier performance; a thicker coating would further increase the lag time at the cost of potentially elevated internal residual stresses that could compromise mechanical stability and adhesion.

The test was conducted in galvanostatic mode, applying a constant anodic current of 9.092 mA (corresponding to a current density of 7.291 mA cm^−2^), which ensures a constant and reproducible rate of hydrogen generation at the entry surface. This condition is essential for attaining a genuine steady state (Iss) and for enabling meaningful comparison between specimens. Furthermore, given that diffusion is a thermally activated process whose rate constant depends exponentially on temperature via the Arrhenius relation, strict temperature control at 20 ± 1 °C was maintained throughout all experiments to ensure that any variation in D_eff_ reflects differences in material properties rather than thermal fluctuations. The use of standardized electrolyte solutions 0.1 M NaOH on the detection side and 0.1 M H_2_SO_4_ on the charging side with the addition of thiourea as a hydrogen recombination poison, ensures consistent electrochemical boundary conditions and suppresses the formation of molecular hydrogen (H_2_) at the entry surface, thereby maximizing the hydrogen absorption efficiency.

The mechanical integrity of the TiN coating throughout the experiment is confirmed by the stable, monotonically rising shape of the permeation transient and the absence of abrupt current discontinuities. The appearance of a sudden, sharp peak in the permeation current would be diagnostic of coating failure by cracking or delamination, as such events would instantaneously expose the underlying steel to direct hydrogen charging. The smooth transient profile observed for the TiN/CS system therefore provides indirect but compelling evidence that the coating remained mechanically intact and fully adherent to the substrate for the entire duration of the experiment.

### 4.4. Comparison with Reference Systems and Literature Context

The diffusion behavior of hydrogen in composite coating/substrate bilayer systems has been addressed analytically by Song et al. [18], who demonstrated that, as coating thickness increases, the through-thickness micropore density decreases while the effective hydrogen diffusivity of the composite increases. This apparently counterintuitive dependence reflects the complex interplay between microstructural evolution and diffusivity and implies that the hydrogen barrier efficiency of a coating cannot be predicted based on thickness alone but must account for the concurrent changes in film microstructure that accompany variations in deposition parameters.

Titanium-based hydrogen permeation barriers primarily consist of binary coatings, such as titanium nitride (TiN) and titanium carbide (TiC), as well as advanced multicomponent composite systems. Among these, TiN coatings are particularly effective due to their ability to significantly inhibit the interstitial diffusion of hydrogen into the metallic substrate. By acting as a dense physical and chemical barrier, these coatings suppress hydrogen ingress, thereby mitigating the risk of embrittlement and enhancing the structural integrity of the base material [3].

In the broader context of nitride-based barrier, TiAlN ternary films have been reported to achieve hydrogen permeabilities in the order of 10^−18^ mol s^−1^ m^−1^ Pa^−1^/^2^ when deposited by magnetron sputtering from Ti and Al targets in a nitrogen-containing atmosphere [19], representing among the lowest values reported for physical vapor deposited coatings. By comparison, the diffusion coefficients determined for palladium-coated API 5L steel specimens have been reported in the range of 1.5 × 10^−11^ to 9.4 × 10^−10^ m^2^ s^−1^ [20], against which the D_eff_ = 2.68 × 10^−10^ m^2^ s^−1^ obtained in the present study for the TiN/CS composite is fully consistent, confirming that the HiPIMS-deposited TiN layer confers a diffusion resistance comparable to established metallic hydrogen-barrier systems.

In summary, the integrated analysis of the permeation data, combined with the mechanistic framework outlined above, demonstrates that the HiPIMS-deposited TiN coating functions not as a merely passive surface layer but as an advanced, multifunctional hydrogen diffusion barrier. Its exceptional performance arises from the synergistic combination of intrinsic low hydrogen permeability, interfacial trapping effects at the TiN/steel boundary, and the high-density, defect-poor microstructure that is a hallmark of the HiPIMS deposition process [21]. These attributes collectively make TiN an attractive and technically robust candidate for protective coatings in hydrogen-rich engineering environments where susceptibility to hydrogen embrittlement must be minimized.

## 5. Conclusions and Future Work

This research focused on the hydrogen permeation barrier performance of a non-commercial TiN coating, which was HiPIMS-deposited on a SAE 1020 carbon steel substrate. Electrochemical permeation measurements were conducted under controlled and standardized experimental conditions in accordance with ASTM G148 [8] and ISO 17081 [9]. Considering the experimental findings and their mechanistic interpretation, these main conclusions emerge.

○ Quantitative validation of barrier performance. The TiN/CS composite system exhibited a lag time of t_lag_ = 570 s, a mean effective diffusion coefficient of D_eff_ = 2.69 × 10^−10^ m^2^ s^−1^ (averaged across four independent time-parameter methods), a steady-state permeation current density of Jss = 21.5 μA cm^−2^, a permeation reduction factor PRF = 2.32, and a barrier efficiency of η = 56.9%. All derived parameters are numerically self-consistent and were independently verified through cross-method calculation, confirming the internal reliability of the experimental dataset.
○ Effective suppression of hydrogen transport kinetics. Relative to the uncoated SAE 1020 reference, the HIPIMS-TiN coating reduced the composite effective diffusion coefficient by 3.8-fold (from D_eff_, CS ≈ 1.03 × 10^−9^ m^2^s^−1^ to D_eff_, TiN/CS = 2.61 × 10^−10^ m^2^s^−1^) and increased the time lag (t_lag_, TiN/CS = 570 s). The hydrogen oxidation current density at steady state decreased from Jss, peak, CS ≈ 50 µA cm^−2^ to Jss, TiN/CS = 21.5 µA cm^−2^, yielding a Permeation Reduction Factor PRFpeak = 2.33 and a barrier efficiency η = 57.1%, in addition to a Permeation Reduction Factor PRFss = 1.09 and a barrier efficiency η = 8.5%. The smooth, monotonic, overshoot-free TiN/CS transient further confirms TiN-limited composite transport and full mechanical coating integrity throughout the 12,000 s experiment.
○ Reduction in steady-state permeation flux. The TiN-coated specimens exhibited a significantly lower steady-state permeation current density (≈21.5 μA cm^−2^) compared with the uncoated carbon steel reference (≈50 μA cm^−2^), corresponding to a reduction of approximately 56% in the hydrogen flux reaching the detection side of the membrane. This consistent reduction in flux, maintained for the entire experimental period, offers quantitative evidence of the coating’s sustained and efficient ability to prevent hydrogen from entering.
○ Significant delay in hydrogen breakthrough. The lag time of 570 s measured for the TiN/CS system represents a substantial retardation of hydrogen entry into the steel substrate relative to the uncoated reference, for which no discernible induction period was observed. This extended induction period is attributed to the combined effect of the intrinsically low hydrogen diffusivity and solubility of the dense TiN crystal lattice, together with the interfacial trapping contribution at the TiN/steel boundary region.
○ Microstructural integrity and coating quality. SEM-EDS analysis confirmed that the HiPIMS-deposited TiN film was dense, compositionally uniform, under the tested conditions. The consistent hydrogen permeation curve for the TiN/CS system indirectly but strongly suggests the coating remained mechanically sound and adhered well during the electrochemical test, showing no cracks or delamination. These microstructural qualities are directly due to the high ionization and energetic deposition inherent to the HiPIMS process
○ Relevance to hydrogen technology applications. The deposition of a TiN thin film onto carbon steel constitutes an effective strategy for mitigating hydrogen-assisted degradation of steel substrates, which is of critical importance for the safe and reliable deployment of hydrogen-based energy technologies. The present results are consistent with the broader literature on nitride-based barrier, which have been identified as promising candidates for improving the hydrogen barrier properties of ferritic steels [19].
○ The bilayer lag-time methodology employed in this work provides a robust and transferable analytical framework for quantifying the hydrogen barrier performance of composite coating–substrate systems, and its application to other alloy families and commercial coating systems represents a natural extension of the present investigation. Furthermore, the research program will be extended to evaluate the performance of hydrogen barrier under hydrogen sulfide (H_2_S) and wet H_2_S service environments, which are highly relevant to upstream oil and gas applications where hydrogen-induced cracking constitutes a critical integrity concern.
○ Scalability and applicability. The present results establish TiN HiPIMS as a viable candidate material for hydrogen barrier applications. Future work should include: (i) high-resolution cross-sectional SEM/TEM for definitive microstructural characterization; (ii) XRD, WDS or XPS to determine Ti:N stoichiometry; (iii) permeation testing under H_2_S and wet H_2_S service conditions, variable-temperature permeation, and triplicate replication of permeation transients; and (iv) evaluation of scalable deposition methods. In addition, quantitative residual stress measurements and surface roughness analysis by contact profilometry, adhesion and Rockwell C testing, thermal TDS, and Pd-overlayer experiments should be carried out for each condition identified.

## Figures and Tables

**Table 1:** Experimental matrix conditions for TiN materials.

Test	Cell	Volume	Reactive
Electrochemical hydrogen permeation	Generation Cell/Reduction	500 mL	CH_4_N_2_S/5g/L
			H_2_SO_4_/0.1 M
			Distilled water
	Sensing Cell/oxidation	500 mL	NaOH/0.1 M
			Distilled water

**Table 2:** Explanation of the parameters used in the analysis of Figure 2 and Figure 3.

Parameter of Analysis	Substrate (CS)	Barrier (TiN + CS)	Correlation and Significance
Permeation Curve Shape	Rapid rise, prominent peak, high plateau.	Slow, delayed rise, no significant peak, low plateau.	Indicates a fundamental change in hydrogen transport from rapid diffusion to a slow, barrier-controlled process.
Peak Current Density	>50 µA/cm^2^	~20 µA/cm^2^	The TiN coating prevents the high initial flux of hydrogen, suggesting it limits hydrogen entry at the surface.
Steady-State Current (Iss)	~0 µA/cm^2^	~20 µA/cm^2^	The steady-state flux through the coated sample is 60% lower, proving a sustained and effective barrier action.
Time to Reach Steady-State	Relatively short (<500 s)	Significantly longer (>8000 s)	Confirms that the TiN layer acts as a diffusion barrier, increasing the time required to establish a steady-state concentration gradient.
Diffusion Coeff. (D)	Higher (typical for steel)	Lower (2.68 × 10^−10^ m^2^/s)	The composite system’s D is dominated by the low-diffusive TiN layer, quantifying the slowing effect on hydrogen transport.
Barrier Efficiency (η)	0% (Baseline)	56%peak/8.5%ss	Provides a clear, quantitative measure of the coating’s effectiveness in preventing hydrogen permeation, relation is a percentage%.

**Table 3:** Data results of Hydrogen permeation of coating TiN + CS.

Variable	Symbol	Value	Units	Description
Sample Thickness	L	0.97	mm	Distance that hydrogen must travel to pass through the sample.
Area of the charged electrode	A_c_	1.246	cm^2^	Total surface area of the electrode where the electric charge is applied.
Area of the detection electrode	A_s_	1.038	cm^2^	Surface area of the electrode where the permeation current is measured. Exposed area of sample in the oxidation cell.
Charging current	I_c_	9.092	mA	Intensity of the electric current applied to introduce hydrogen into the sample.
Charging current density	I_c’_	7.291	mA/cm^2^	Charging current per unit area.
Start time of charging	t_0_	3665	s	Time at which the application of the charging current begins.
Initial current	I_0_	−5.38 × 10^−7^	A	Value of the current before applying the charge.
Limiting current	I_sat_	0.02153	mA/cm^2^	Maximum value of the permeation current reached.
Saturation concentration	C_sat_	8.063; 1.02	mol H/m^3^; ppm	Maximum concentration of hydrogen can be dissolved in the material.
Time lag	t_lag_	570	s	Time taken for the permeation current to reach half of its steady state value. J(t)/J_ss_ = 0.63, t_lag_ = L^2/^6*D_app_, D = L^2^/(6 × t_lag_) (7)
Mean time	t_1/2_	465	s	The average time for hydrogen atoms to diffuse through the material, is the time to reach 50% of the steady-state current.
Advance time	t_b_	233	s	Time corresponding to the point of inflection in the permeation curve. elapsed time measured extrapolating the linear portion of the rising permeation current transient to J(t) = O(s)
Inflection time	t_i_	345	s	Time at which the permeation curve changes its curvature.
Diffusion coefficient	D_eff_	2.68 × 10^−10^	m^2^/s	The rate at which hydrogen atoms diffuse through the material sample. Average diffusion coefficient.
Diffusion coefficient (t_lag_)	D (t_lag_)	2.75 × 10^−10^	m^2^/s	Time taken for the permeation current to reach a steady value and the diffusion coefficient of hydrogen in the material, respectively.
Diffusion coefficient (t_1/2_)	D(t_1/2_)	2.79 × 10^−10^	m^2^/s	Diffusion coefficient of hydrogen in the material, t _1/2_.
Diffusion coefficient (t_b_)	D(t_b_)	2.64 × 10^−10^	m^2^/s	Diffusion coefficient of hydrogen in the material, t_b_. D = L^2^/(15.3 × t_b_) (8)
Diffusion coefficient (t_i_)	D(t_i_)	2.55 × 10^−10^	m^2^/s	Diffusion coefficient of hydrogen in the material, t_i_.

## Data Availability

The original contributions presented in this study are included in the article. Further inquiries can be directed to the corresponding author.
